# Core-Shell-Like Structured Co_3_O_4_@SiO_2_ Catalyst for Highly Efficient Catalytic Elimination of Ozone

**DOI:** 10.3389/fchem.2021.803464

**Published:** 2021-12-09

**Authors:** Jingya Ding, Feng Cheng, Zhen Meng, Yan Cao, Fennv Han, Dongbin Chen, Mingxiang Cao, Guolin Zhang, Jiahao Kang, Shuxiang Xu, Qi Xu

**Affiliations:** ^1^ School of Chemistry and Chemical Engineering, Yancheng Institute of Technology, Yancheng, China; ^2^ Key Laboratory for Advanced Technology in Environmental Protection of Jiangsu Province, Yancheng Institute of Technology, Yancheng, China; ^3^ School of Mechanical Engineering, Yancheng Institute of Technology, Yancheng, China

**Keywords:** ozone decomposition, Co_3_O_4_, SiO_2_, Core@Shell constructure, relative humidity 1. introduction

## Abstract

Co_3_O_4_ is an environmental catalyst that can effectively decompose ozone, but is strongly affected by water vapor. In this study, Co_3_O_4_@SiO_2_ catalysts with a core-shell-like structure were synthesized following the hydrothermal method. At 60% relative humidity and a space velocity of 720,000 h^−1^, the prepared Co_3_O_4_@SiO_2_ obtained 95% ozone decomposition for 40 ppm ozone after 6 h, which far outperformed that of the 25wt% Co_3_O_4_/SiO_2_ catalysts. The superiority of Co_3_O_4_@SiO_2_ is ascribed to its core@shell structure, in which Co_3_O_4_ is wrapped inside the SiO_2_ shell structure to avoid air exposure. This research provides important guidance for the high humidity resistance of catalysts for ozone decomposition.

## Introduction

Ozone is widely used in food, medicine, and waste treatment owing to its excellent oxidizing ability (Alameddine et al., 2021; Kim et al., 2022). However, even low concentrations of ozone are harmful to human health, especially to the eyes, nose, and throat (Ferrara et al., 2020; Ferrara et al., 2021). The maximum eight-hour average concentration of ozone allowed by the World Health Organization is 100 μg/m^3^. Ozone concentrations in the atmosphere near ground level have considerably increased in recent years due to increased levels of volatile organic compounds and nitrogen oxides (Ou et al., 2016). Ozone in the outdoor air can infiltrate into indoor environments. Indoor ozone is considered more harmful than outdoor ozone because modern humans spend most of their time indoors (Abbass et al., 2017; Namdari et al., 2021; Nazaroff and Weschler 2021). The development of environmental technologies to effectively eliminate ozone is therefore necessary.

There are four common treatment methods to eliminate ozone: activated carbon (Yu et al., 2020); absorption (Yang et al., 2017); thermal decomposition and catalytic decomposition (Gopi et al., 2017; Ma et al., 2017; Gong et al., 2018). Catalytic decomposition is considered to be one of the most feasible and effective methods for ozone removal (Li et al., 2020). Noble metals and transitional metal oxides are common catalysts for heterogeneous reactions including decomposition of ozone (Nikolov et al., 2010; Gong et al., 2017; Deng et al., 2019; Tao et al., 2021a; Tao et al., 2021b). Among the transition metal oxides, Co_x_O_y_ catalysts with higher oxidation states have exhibited higher ozone decomposition performance than other cobalt oxide catalysts (Tang et al., 2014a). Abdedayem (Abdedayem et al., 2017) demonstrated that the ozone decomposition abilities of Co_3_O_4_ support on loaded olivine is proportional to its dispersion degree. However, numerous metal oxide catalysts suffer from interactions with water vapor, and including cobalt oxides (Zhu et al., 2021). It is generally believed that water vapor affects ozone decomposition via competitive adsorption with the transition metal oxides on the active sites (Jia et al., 2016).

In this study, core@shell structure catalysts were synthesized with mesoporous silica as the shell and Co_3_O_4_ nanoparticles as the core (Co_3_O_4_@SiO_2_) following the solvothermal route using polyvinylpyrrolidone (PVP) as the capping agent. For comparison, spherical silica supported different additions of Co_3_O_4_ and labeled xCo_3_O_4_/SiO_2_, where x = 10, 15, and 20, or 25%. The ozone decomposition performance of xCo_3_O_4_/SiO_2_ increased with increasing Co_3_O_4_. The 25Co_3_O_4_/SiO_2_ and Co_3_O_4_@SiO_2_ catalysts yielded high ozone decomposition activity at 20% relative humidity. The ozone elimination activity of 20Co_3_O_4_/SiO_2_ sharply decreased upon increasing the relative humidity to 60%, and the Co_3_O_4_@SiO_2_ catalyst exhibited a better moisture resistance performance for ozone decomposition. This study provides important insights for the further development of coated catalysts for gaseous ozone decomposition.

## Experimental Methods

### Catalyst Preparation

Co_3_O_4_@SiO_2_ was synthesized in accordance with previously published studies (Khan et al., 2015). First, 0.70 g PVP and 0.35 g Co(NO_3_)_2_·6H_2_O were dissolved in 40 ml ethanol. The solutions were transferred to stainless steel lined with Poly tetra fluoroethylene (PTFE) in an autoclave and heated at 453 K for 4 h. The obtained black powder was dispersed in 103.8 ml ethanol, to which 82.8 ml distilled water, 7.2 ml 25% aqueous ammonia solution, 0.3 g cetyltrimethylammonium bromide, and 1.0 ml tetraethoxysilane were added. The solution was stirred for 48 h at room temperature. The product was collected via filtration, washed three times with distilled water, dried at 333 K, and then calcined at 773 K for 6 h. The finished samples were denoted as Co_3_O_4_@SiO_2_ (wt% = 30%). SiO_2_ was impregnated with 10, 15, 20, or 25% cobalt loading in an ethanol solution of cobalt nitrate, and the resulting product was calcined at 773 K for 6 h. The prepared samples were labeled as 10Co_3_O_4_/SiO_2_, 15Co_3_O_4_/SiO_2_, 20Co_3_O_4_/SiO_2_, and 25Co_3_O_4_/SiO_2_, respectively.

### Catalyst Characterization

The samples were characterized by X-ray diffraction (XRD) using a D/max-RB diffractometer. X-ray photoelectron spectroscopy (XPS) was performed using a Thermo Fisher ESCALAB 250Xi. Morphological and microstructural characterizations were carried out using a Hitachi EM-3010 transmission electron microscope (TEM). The surface areas were calculated by the Brunauer-Emmett-Teller (BET) method. The pore diameters were estimated from the desorption branchers of the isotherms based on the Barrett-Joyner-Halenda (BJH) model.

### Catalyst Test

The ozone decomposition activity of the prepared catalysts was evaluated using a flow-through quartz tube reactor (inner diameter = 10 mm) with 0.10 g of catalyst separated by quartz sand at different temperatures and relative humidity (20, 40, and 60%) under atmospheric pressure conditions. Ozone was generated by flowing 20% O_2_/N_2_ compressed gas through an ozone generator. The relative humidity of the gas stream was measured using a humidity probe (Benetech, GM1361+). The total gas flow rate passing through the quartz reactor was controlled at 1,500 ml/min and contained 40 ppm O_3_. The ozone concentrations at the inlet and outlet were detected using a 106-L ozone online analyzer (2B Technologies, Boulder, Co, United States). The ozone conversion was calculated according to:

$$\text{Ozone conversion}=\frac{C_{i}\left(O_{3}\right)-C_{o}\left(O_{3}\right)}{C_{i}\left(O_{3}\right)}\times 100\text{\%}$$

where $c_{i}\left(O_{3}\right)$ and $c_{o}\left(O_{3}\right)$ represent the inlet and outlet ozone concentrations, respectively.

## Results and Discussion

### Catalyst Characteristics

**FIGURE 1:**
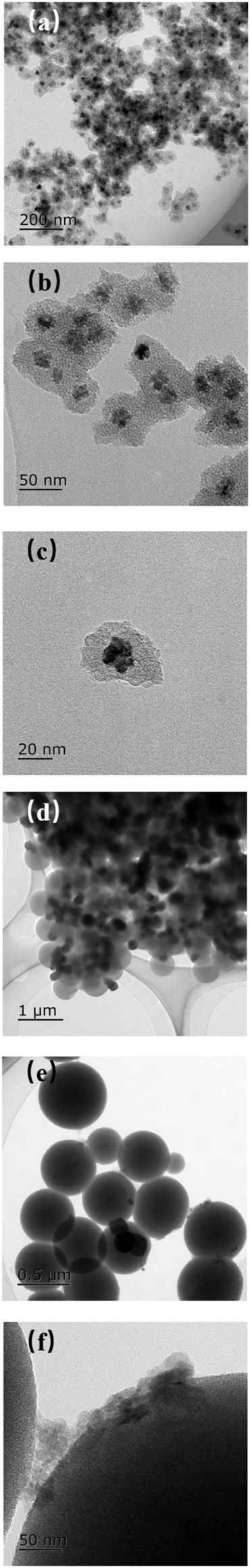
Scanning electron microscope images of Co_3_O_4_@SiO_2_ **(A–C)** and 25Co_3_O_4_/SiO_2_ **(D–F)**.

The morphology and nanostructure of the catalysts were observed by TEM. Figure 1A–C show that the Co_3_O_4_@SiO_2_ nanoparticles were relatively dispersible with an average size of 40 nm. This indicates that PVP can prevent Co_3_O_4_ nanoparticle agglomeration under hydrothermal conditions. Figure 1D–F show that the spherical Co_3_O_4_/SiO_2_ composites prepared via incipient wetness impregnation were highly dispersed with a relatively smooth external surface. This indicates that a majority of the Co_3_O_4_ nanoparticles were incorporated into the mesopores (Xie et al., 2011). With regard to the spent Co_3_O_4_/SiO_2_ catalyst, the large aggregates were clearly located on the external surface of the spherical SiO2 support, and indicating that small Co_3_O_4_ nanoparticles outside of the mesopores easily agglomerated into large Co_3_O_4_ aggregates during the reaction.

**FIGURE 2:**
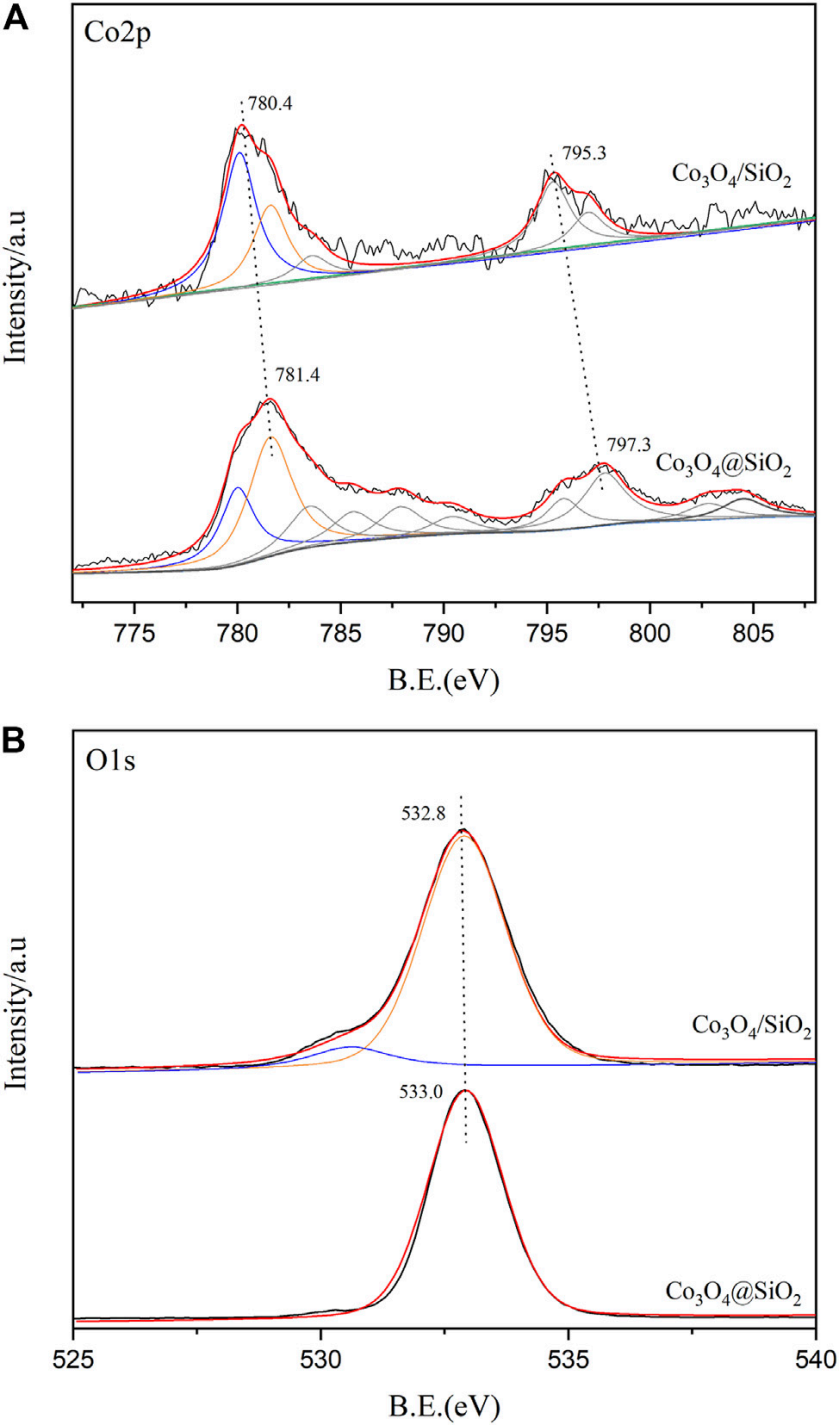
XPS survey spectra of Co_3_O_4_@SiO_2_ and 25Co_3_O_4_/SiO_2_.

X-ray photoelectron spectroscopy (XPS) tests were performed to detect the chemical state and composition of the element catalyst surface. According to the previously reported Co_3_O_4_ spectrum (Gao et al., 2021), the Co 2p spectrum of Co_3_O_4_ (Figure 2A) consists of two peaks, Co 2p_3/2_ and Co 2p_1/2_, located at 779.9 and 794.8 eV, respectively. However, the Co 2p_3/2_ and Co 2p_1/2_ peaks in the Co_3_O_4_@SiO_2_ and Co_3_O_4_/SiO_2_ catalysts shifted to approximately 781.0 and 796.0 eV, respectively, both of which occur at higher energies than those of pure Co_3_O_4_. This shift is mainly due to the interaction between the silica and Co_3_O_4_ species, which results in a charge transfer from the Co_3_O_4_ to the SiO_2_ support and has a positive impact on the cobalt catalytic performance.

The atomic surface contents of cobalt were 0.8 and 7.6% for the Co_3_O_4_@SiO_2_ and Co_3_O_4_/SiO_2_ catalysts, respectively. This significant difference further confirms that the preparation of Co_3_O_4_@SiO_2_ successfully encapsulated Co_3_O_4_ into the SiO_2_ matrix. The O 1 s spectra of the catalysts are shown in Figure 2B. The main O 1 s peak centered at 533.0 eV represents the lattice oxygen of Co_3_O_4_ and SiO_2_, but is difficult to be accurately distinguished. The oxygen in the unreducible silica has no notable effect on the catalysis of ozone.

**FIGURE 3:**
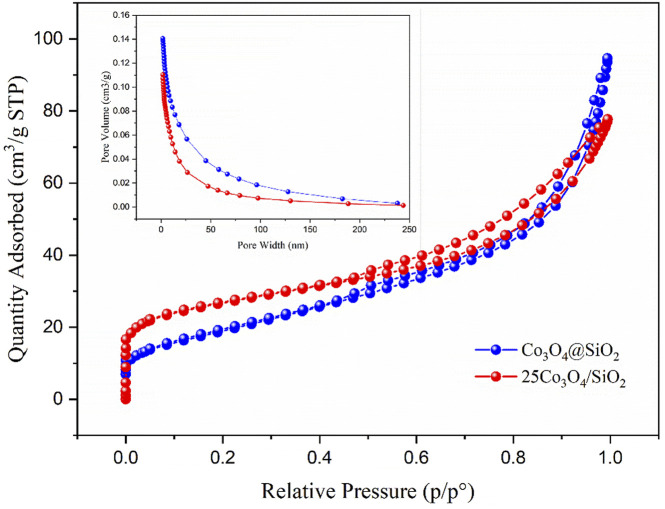
Nitrogen adsorption-desorption isotherms and BJH pore-size distribution curves of Co_3_O_4_@SiO_2_ and 25Co_3_O_4_/SiO_2_.

Figure 3 shows the nitrogen isothermal adsorption-desorption curves and pore size distributions of the Co_3_O_4_@SiO_2_ and 25Co_3_O_4_/SiO_2_ catalysts. The nitrogen adsorption-desorption isotherms clearly show that both samples have typical hysteresis loops and are classified as type-IV isotherms, thus indicating that the samples have a mesoporous structure. The average pore diameter of the two samples ranges between 6 and 9 nm. The pore volume of Co_3_O_4_@SiO_2_ (0.15 cm^3^/g) is larger than that of Co_3_O_4_/SiO_2_ (0.11 cm^3^/g). The specific surface area of Co_3_O_4_/SiO_2_ is 94.8 m^2^/g, which is 1.5 times greater than that of Co_3_O_4_@SiO_2_ (68.8 m^2^/g). The specific surface area of a catalyst is generally believed to have a substantial impact on the catalytic activity, in which catalysts with larger specific surface areas usually have higher catalytic activities. Effect of Co_3_O_4_@SiO_2_ and Co_3_O_4_/SiO_2_ on ozone decomposition.

**FIGURE 4:**
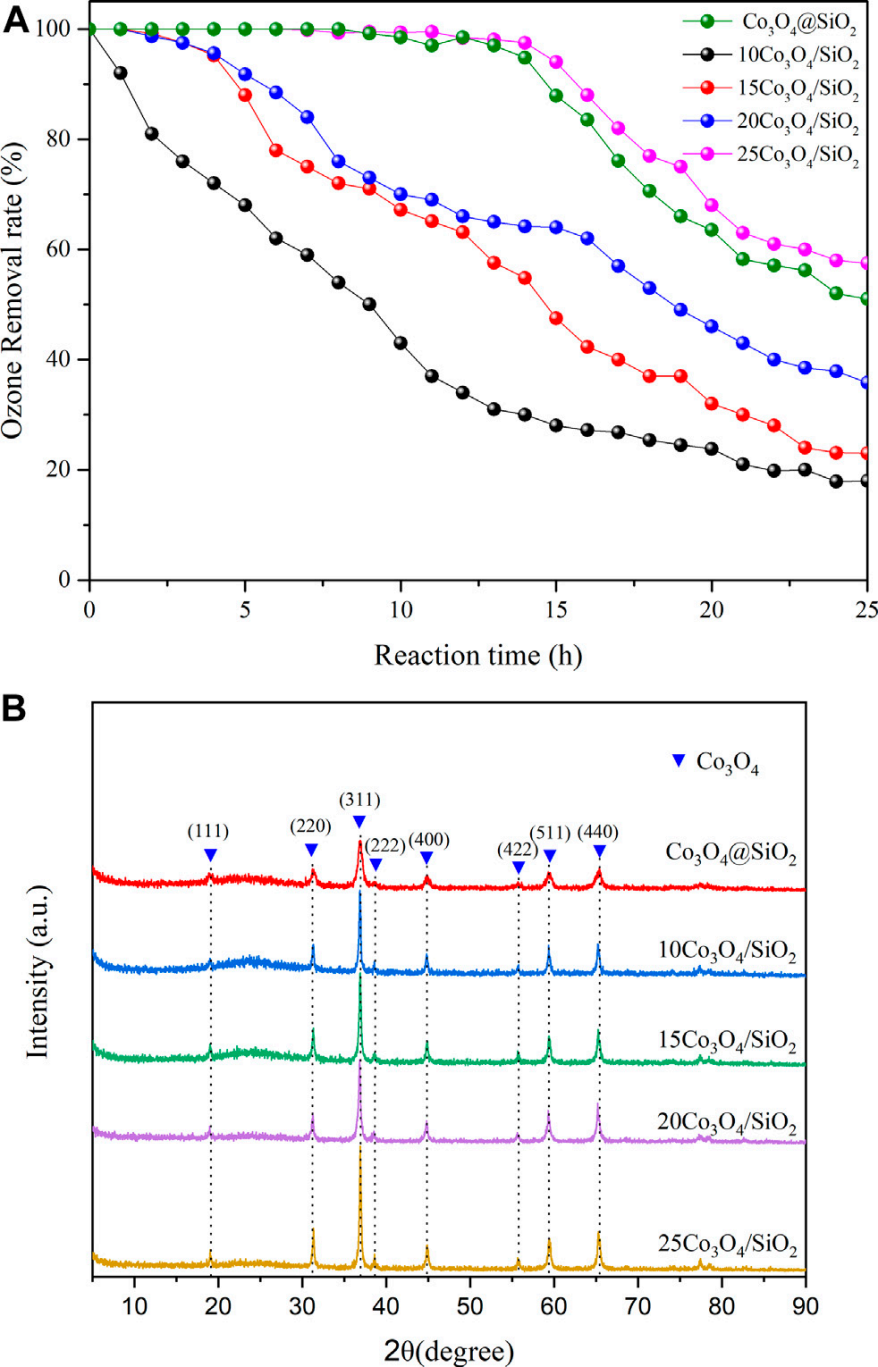
**(A)** Ozone removal rates and **(B)** XRD patterns of Co_3_O_4_@SiO_2_ and xCo_3_O_4_ (x = 10, 15, 20, and 25). Temperature = 25°C; relative humidity = 20%.

The ozone decomposition rates of Co_3_O_4_@SiO_2_ and Co_3_O_4_/SiO_2_ with different Co_3_O_4_ loadings were evaluated in a gas flow with 40 ppm ozone at 20% relative humidity. The activity of the 10Co_3_O_4_/SiO_2_ catalyst dropped sharply within 1 h, and the 15Co_3_O_4_/SiO_2_ and 20Co_3_O_4_/SiO_2_ catalysts dropped to 94% ozone conversion after 4 h. The time to achieve 100% ozone removal rate increased to 9 h for a Co_3_O_4_ load of 25%. However, the Co_3_O_4_@SiO_2_ catalyst with 30   wt% loading achieved the same ozone removal rate as that of 25Co_3_O_4_/SiO_2_. This indicates that the ozone elimination rate is proportional to the Co_3_O_4_ catalyst load. The XRD patterns of the as-prepared catalysts are shown in Figure 4B, in which all of the obtained samples exhibit the same peaks, corresponding to pure Co_3_O_4_ (JCPDS No. 42-1,467) (Agilandeswari and Rubankumar 2016). This indicates that the crystalline phase is well maintained during the treatment. The diffraction peaks of both Co_3_O_4_@SiO_2_ and xCo_3_O_4_/SiO_2_ are sharp and intense, and the peak intensities gradually increase with increasing Co_3_O_4_ catalyst load. The 25Co_3_O_4_/SiO_2_ catalyst exhibits more intense peaks at 36.5 than Co_3_O_4_@SiO_2_ for a similar Co_3_O_4_ content. This indicates that the Co_3_O_4_@SiO_2_ core-shell structure weakens the intensity of the characteristic peaks, and that the Co_3_O_4_ crystalline material is well inside the mesoporous silica particles. Effect of 25Co_3_O_4_@SiO_2_ and Co_3_O_4_/SiO_2_ on ozone decomposition under different relative humidity conditions.

**FIGURE 5:**
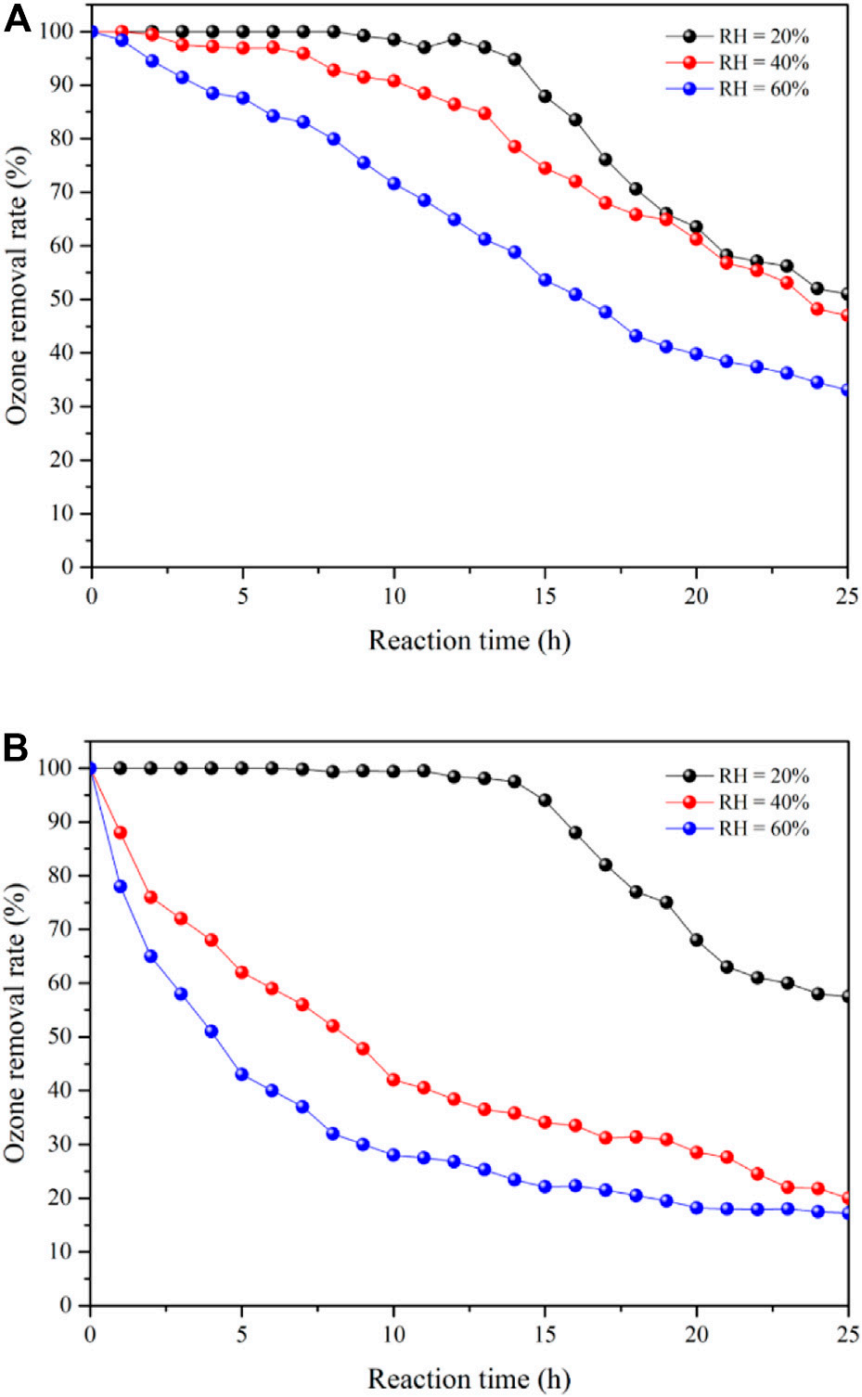
Ozone removal rate of 25Co_3_O_4_@SiO_2_ **(A)** and Co_3_O_4_/SiO_2_ **(B)**. Temperature = 25°C; relative humidity (RH) = 20, 40, and 60%.

Figure 5A,B show the ozone removal rates of 25Co_3_O_4_@SiO_2_ and Co_3_O_4_/SiO_2_, respectively, at relative humidity conditions of 20, 40, and 60%. The 25Co_3_O_4_@SiO_2_ and Co_3_O_4_/SiO_2_ catalysts exhibit similar ozone removal rates at 20% relative humidity. The 25Co_3_O_4_/SiO_2_ catalyst shows 99% ozone conversion for 11 h at 20% relative humidity. The removal rate then sharply drops and ultimately stabilizes at 60%. It is noted that the ozone removal rate sharply decreases with increasing relative humidity, especially when the relative humidity is increased from 40 to 60%. For Co_3_O_4_@SiO_2_, the ozone removal rate begins to decrease during the first 12 h of the reaction runs with a gas flow of 40 ppm ozone, and then decreases to 60% when the reaction has been maintained for 24 h. The ozone removal rate of Co_3_O_4_@SiO_2_ shows a different trend from that of 25Co_3_O_4_/SiO_2_ at 40% relative humidity. When the relative humidity is increased to 60%, the ozone removal rate sharply decreases and remains at 30%, which is approximately 10% higher than that of 25Co_3_O_4_/SiO_2_. This indicates that the main reason for the different performance of the two catalysts is their differing structures. The Co_3_O_4_ loaded on the surface of SiO_2_ is directly exposed to the reaction environment. The accumulation of oxygen atoms and adsorption of water vapor thus lead to catalyst deactivation. In contrast, in the Co_3_O_4_@SiO_2_ catalyst, the Co_3_O_4_ is wrapped by SiO_2_, and which isolates water vapor and prevents it from directly contacting with the Co_3_O_4_. The deactivation can thus be attributed to the accumulation of oxygen atoms.

### Proposed Mechanism

According to the experimental results, we proposed a possible mechanism involving oxygen vacancies (O_v_) as depicted below. Initially, the ozone molecule is adsorbed on the oxygen vacancy of the surface of Co_3_O_4_ and the ozone decompose into oxygen, while another oxygen atom is left on the surface of Co_3_O_4_ and form lattice oxygen (O^2−^). Subsequently, the ozone molecule reacts with lattice oxygen and form oxygen and O_2_^2−^. Finally, the O_2_^2−^ breaks off the Co_3_O_4_ surface in form of oxygen.

$$O_{3}+O_{V}\to O_{2}+O^{\mathrm{2-}}\;\;\left(R1\right)$$

$$O_{3}+O^{\mathrm{2-}}\to O_{2}+O_{2}^{\mathrm{2-}}\;\;\left(R2\right)$$

$$O_{2}^{\mathrm{2-}}\to O_{2}+O_{v}\;\;\left(R3\right)$$

## Conclusion

In this work, Co_3_O_4_@SiO_2_ and xCo_3_O_4_/SiO_2_ (x = 10, 15, 20, and 25) catalysts were successfully synthesized using the hydrothermal method. Under similar loading conditions, the ozone removal rates of Co_3_O_4_@SiO_2_ and 25Co_3_O_4_/SiO_2_ were nearly the same under flow conditions of 40 ppm ozone and 20% relative humidity. When the relative humidity increased to 60%, the ozone removal rate of Co_3_O_4_@SiO_2_ was higher than that of 25Co_3_O_4_@SiO_2_. XRD, XPS, and BET characterizations indicate that the high Co_3_O_4_@SiO_2_ performance is related to the core@shell structure. This study thus provides insight for developing catalysts to effectively remove gaseous ozone.

## Data Availability

The original contributions presented in the study are included in the article/Supplementary Material, further inquiries can be directed to the corresponding authors.
