# Supplementary material for: Core-Shell-Like Structured Co3O4@SiO2 Catalyst for Highly Efficient Catalytic Elimination of Ozone
Source: Front Chem. 2021 Dec 9;9:803464. doi: 10.3389/fchem.2021.803464 (PMC8695612; doi:10.3389/fchem.2021.803464)
Supplement: Supplementary file 2 [file Table1.DOC]

**Core-shell-like structured Co_3_O_4_@SiO_2_ catalyst for highly efficient catalytic elimination of ozone**

Jingya Ding^1^, Feng Cheng^2*^, Zhen Meng^1^, Yan Cao^3^, Fennv Han^1^, Dongbin Chen^1^, Mingxiang Cao^1^, Guolin Zhang^3^, Jiahao Kang^1^, Shuxiang Xu^1^, and Qi Xu^1*^

^1^School of Chemistry and Chemical Engineering, Yancheng Institute of Technology, China

^2^Key Laboratory for Advanced Technology in Environmental Protection of Jiangsu Province, Yancheng Institute of Technology, China

^3^School of Mechanical Engineering, Yancheng Institute of Technology, Yancheng China





Figure S1. The crystal structure of core-shell structured Co_3_O_4_@SiO_2_ and Co_3_O_4_/SiO_2_ was identified by the XRD. The diffraction peaks of Co_3_O_4_@SiO_2_ and Co_3_O_4_/SiO_2_ can be indexed to the monoclinic phase of Co_3_O_4_（JCPDS No. 42-1467. Some diffraction peaks of the crystalline Co_3_O_4_ are present at 2θ = 19.0^o^、31.3^o^、36.8^o^、38.5^o^、44.8^o^、55.6^o^、59.4^o^ and 65.2^o^, corresponding to the (111)、(220)、(311)、(222)、(400)、(422)、(511) and (440) reflections, respectively. The diffraction peaks in relation to silicon are not observed, which probably exists in the amorphous form. Compared with the standard card, the diffraction peaks are not observed to shift, which means that the Co_3_O_4_/SiO_2_ solid solution phase is not formed.





Figure S2. The X-ray photoelectron spectroscopy (XPS) test was performed to detect the chemical state and composition of the element catalyst surface. Meanwhile, the surface atomic content of cobalt was 0.8% and 7.6% for Co_3_O_4_@SiO_2_ and Co_3_O_4_/SiO_2_ catalysts, respectively. The significant difference in surface content further confirmed that the preparation of Co_3_O_4_@SiO_2_, which encapsulated Co_3_O_4_ into the SiO_2_ matrix successfully.


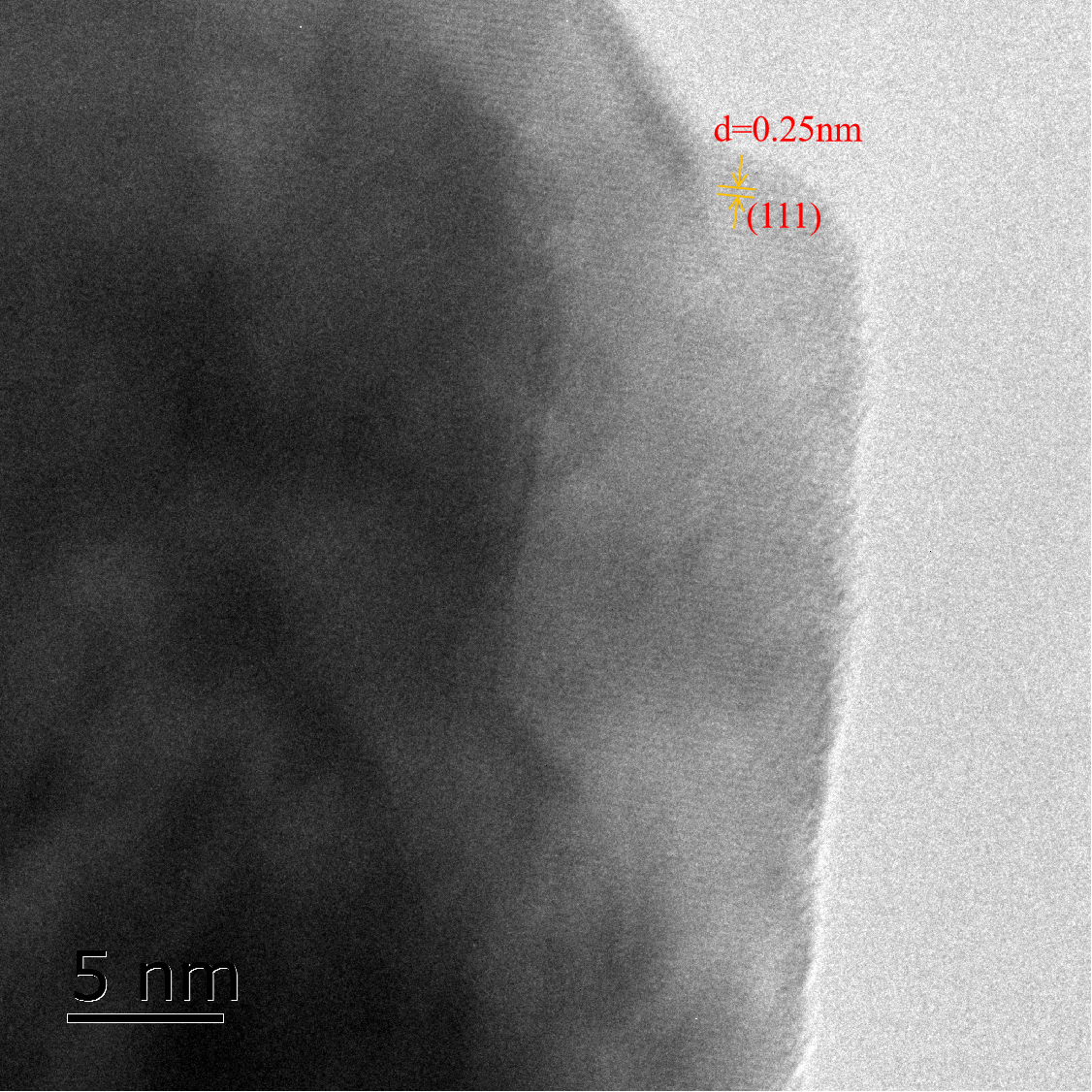


Figure S3. The HRTEM image of core-shell-shell structured Co_3_O_4_/SiO_2_ shows that the lattice spacing is 0.25 nm, corresponding to the (111) plane of the monoclinic phase Co_3_O_4_.
